# Are Compression Stockings an Effective Treatment for Orthostatic Presyncope?

**DOI:** 10.1371/journal.pone.0028193

**Published:** 2011-12-16

**Authors:** Clare Louise Protheroe, Anastasia Dikareva, Carlo Menon, Victoria Elizabeth Claydon

**Affiliations:** 1 Department of Biomedical Physiology and Kinesiology, School of Engineering Science, Simon Fraser University, British Columbia, Canada; 2 Menrva Research Group, School of Engineering Science, Simon Fraser University, British Columbia, Canada; University of Modena and Reggio Emilia, Italy

## Abstract

**Background:**

Syncope, or fainting, affects approximately 6.2% of the population, and is associated with significant comorbidity. Many syncopal events occur secondary to excessive venous pooling and capillary filtration in the lower limbs when upright. As such, a common approach to the management of syncope is the use of compression stockings. However, research confirming their efficacy is lacking. We aimed to investigate the effect of graded calf compression stockings on orthostatic tolerance.

**Methodology/Principal Findings:**

We evaluated orthostatic tolerance (OT) and haemodynamic control in 15 healthy volunteers wearing graded calf compression stockings compared to two placebo stockings in a randomized, cross-over, double-blind fashion. OT (time to presyncope, min) was determined using combined head-upright tilting and lower body negative pressure applied until presyncope. Throughout testing we continuously monitored beat-to-beat blood pressures, heart rate, stroke volume and cardiac output (finger plethysmography), cerebral and forearm blood flow velocities (Doppler ultrasound) and breath-by-breath end tidal gases. There were no significant differences in OT between compression stocking (26.0±2.3 min) and calf (29.3±2.4 min) or ankle (27.6±3.1 min) placebo conditions. Cardiovascular, cerebral and respiratory responses were similar in all conditions. The efficacy of compression stockings was related to anthropometric parameters, and could be predicted by a model based on the subject's calf circumference and shoe size (r = 0.780, p = 0.004).

**Conclusions/Significance:**

These data question the use of calf compression stockings for orthostatic intolerance and highlight the need for individualised therapy accounting for anthropometric variables when considering treatment with compression stockings.

## Introduction

Syncope, or fainting, is described as a transient loss of consciousness and postural tone, with spontaneous recovery [1]. It typically occurs when upright and is associated with reduced cerebral blood flow, often attributed to sudden onset hypotension and bradycardia, associated with the “vasovagal” response [2].

Many syncopal events are triggered by orthostatic stress, likely due to concomitant venous pooling and enhanced capillary filtration when upright [1]. This reduces venous return and, if not adequately compensated, leads to profound reductions in blood pressure and cerebral blood flow [1].

The prevalence of syncope is high, with presyncope and orthostatic dizziness reported in 12.5% of individuals [3], and 0.9–5% of emergency visits and 1% of hospital admissions due to syncopal episodes [4]–[8]. Furthermore, syncope and presyncope have a marked negative impact on quality of life, with many individuals reporting injury secondary to an associated fall or accident during the event; recurrent episodes are particularly debilitating [3], [9]–[11].

The treatment of orthostatic syncope can be particularly challenging. Usually the initial approach is patient counselling [10], [11] incorporating avoidance of known triggers, encouraging adequate hydration (often with salt supplementation) [12], [13], and physical countermanouvres [14]. While these strategies aid in the management of occasional syncope, they are not usually sufficient for the treatment of frequent or severe episodes [11]. Additional treatment strategies include cardiac pacemakers for syncope with cardioinhibition, and pharmacologic therapy, although their utility and efficacy has been questioned [15], [16].

The use of compression hosiery is commonly recommended for those affected by recurrent orthostatic intolerance, based on the rationale that external counter-pressure of the lower limbs or abdomen will reduce venous pooling and capillary filtration, thereby increasing venous return and preventing or delaying the onset of syncope [17]–[19]. Certainly, pooling and filtration in the legs can be extensive, with 500 ml of blood lost into the legs within just 10 minutes of 60° head-upright tilting [20]; therefore, the potential to ameliorate this effect using leg compression garments might be expected to have a profound impact on orthostatic tolerance. Graduated compression garments are thought to be most effective for the treatment of orthostatic syncope, because the movement of body fluid when upright redistributes hydrostatic pressures throughout the body, with the highest pressures found at the ankles [21]. Thus, garments designed to apply greater counter-pressures at the extremities might be expected to be more efficacious. However, despite the common recommendation for patients with orthostatic intolerance to utilise compression stockings [10], [16], there is little research proving their efficacy.

Short term improvements in orthostatic blood pressures with garments applying counter-pressure to the whole leg and/or abdominal segments have been reported [22]–[26], although compression of the thighs may promote venous pooling when sitting, due to a reversal of the pressure gradient [27], with an expected deleterious effect on orthostatic tolerance (OT). Generally, garments that compress the abdomen show greater promise for the prevention of orthostatic intolerance [24]–[26]. However, these are reported to be uncomfortable, difficult to put on and remove, and are associated with poor patient compliance [28], [29].

We aimed to evaluate whether graded calf compression stockings increase OT using a randomised, placebo-controlled, double-blind design. We evaluated calf-high compression stockings so that, if effective, there would be higher compliance and garment comfort for the target patient population [28]. We hypothesized that graded calf compression stockings would improve OT during a progressive orthostatic stress test consisting of combined head-upright tilting and lower body negative pressure (LBNP) [30], [31].

## Methods

### Ethics statement

Ethical approval was obtained from the Simon Fraser University Research Ethics Board and experiments were conducted in accordance with the Declaration of Helsinki. All subjects provided written informed consent.

### Study design

Fifteen adults (six females; aged 25.5±1.3 years) were recruited for this study. Prior to testing subjects completed a brief medical history; all volunteers were healthy and free of cardiovascular and neurological disease. None of the volunteers was taking any medication, except for three females who were using oral contraceptives. Each subject completed testing on three separate days within a week, or at monthly intervals, wearing each of three different types of stocking: calf-length graded compression stocking (Knee-high Graded Support Therapy Socks, Sigvaris Inc, Peachtree City, USA); standard calf-length socks not designed to provide compression, but visually similar to the compression stocking (calf placebo); and ankle-length socks that did not compress the calf (ankle placebo). Testing was conducted in a randomised double-blind fashion, at the same time of day (in the morning). Female subjects were tested in the same phase of their menstrual cycle, achieved by testing on either consecutive days, or at monthly intervals. Subjects were asked to refrain from strenuous exercise twelve hours prior to each test, eat a light breakfast, and avoid caffeine on the morning of each test.

Prior to testing, anthropometric measures were taken. Circumference and skinfold thickness of the calf were determined using a standard tape measure and skinfold callipers (Slim Guide®, Creative Health Products, Plymouth, USA) at the widest level of the calf. Measures were taken in triplicate on the right leg, and the average used for analysis. Calf cross-sectional area (cm^2^) was estimated from the circumference (cm), assuming circularity [32]:
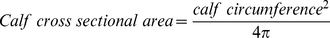



Measures of circumference and skinfold thickness (cm) were used to calculate subcutaneous adipose tissue cross-sectional area (cm^2^):
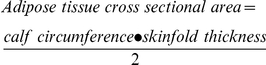



Muscle cross-sectional area (cm^2^), also assumed to be circular, was estimated by the difference between cross-sectional area of the whole limb and adipose tissue with an assumed cross-section of bone with its constituent marrow (6 cm) [32]:
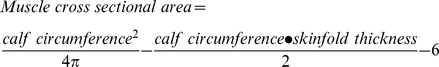



To account for leg shape, height, and foot size, calf circumference was expressed as ratios relative to the subject's height and shoe size.

### Test protocol

On each test day subjects completed an orthostatic stress test consisting of combined head-upright tilting and graded LBNP [30], [31]. After twenty minutes of supine rest, they were tilted to 60°, for twenty minutes. This was followed by incremental increases in LBNP at −20 mmHg, −40 mmHg and −60 mmHg for ten minutes each. The test was terminated if either: their systolic blood pressure fell below 80 mmHg; their heart rate (HR) was less than 50 bpm or greater than 180 bpm; they experienced presyncopal symptoms such as light-headedness, nausea, perspiration and warmth; or the entire protocol was completed. At test termination, the tilt table was rapidly returned to the supine position. OT was taken as the time to presyncope in minutes, from the start of tilting until the test was terminated.

Throughout testing we continuously recorded non-invasive beat-to-beat finger arterial pressures (Finometer, Finapres Medical Systems, Amsterdam, The Netherlands). This device also calculates beat-to-beat cardiac output (CO), stroke volume (SV), and total peripheral resistance (TPR), using the Modelflow technique [33]–[35]. HR and rhythm were monitored using a lead II electrocardiogram (ECG; Finapres ECG Module, Finapres Medical Systems, Amsterdam, The Netherlands). We also monitored the partial pressures of end tidal oxygen (P_ET_O_2_) and carbon dioxide (P_ET_CO_2_) on a breath-by-breath basis (O_2_Cap Oxygen Analyser, Oxigraph Inc, California, USA). Mean cerebral blood flow velocity (CBFV) in the middle cerebral artery was measured using a 2 MHz ultrasound probe located at the right temporal window and secured in position using a headband; similarly, brachial artery blood flow velocity was measured with an 8 MHz ultrasound probe held in place over the brachial artery by an adjustable clamp, with the arm supported at heart level (Doppler Box, Compumedics Germany GmbH, The DWL Doppler Company, Singen, Germany). Data acquisition was performed with a sampling frequency of 1 KHz using an analog-to-digital converter (Powerlab 16/30, AD Instruments, Colorado Springs, CO).

### Data analysis

Mean arterial pressure (MAP) was calculated as diastolic arterial pressure +1/3 pulse pressure. Forearm vascular resistance (FVR) was calculated as MAP divided by brachial blood flow velocity. Cerebral mean arterial pressure (CMAP) was calculated from MAP at heart level, corrected for the measured height difference between the temporal window and heart when upright [36]. Cerebrovascular resistance (CVR) was taken as CMAP/CBFV. The efficacy of cerebral autoregulation was quantified from the correlation coefficient and gradient describing the relationship between CMAP and CBFV, as described previously [36]. Data are presented as 30 second averages, every two minutes throughout testing. Data at presyncope represent the final value for each variable prior to the return to the supine position. Note that because of variable times at which presyncope was initiated and the tests stopped, the number of subjects included for each data point decreased as the test progressed. Thus, cardiovascular responses are presented only for the first 30 minutes of orthostasis.

### Measures of calf compression

**Figure 1:**
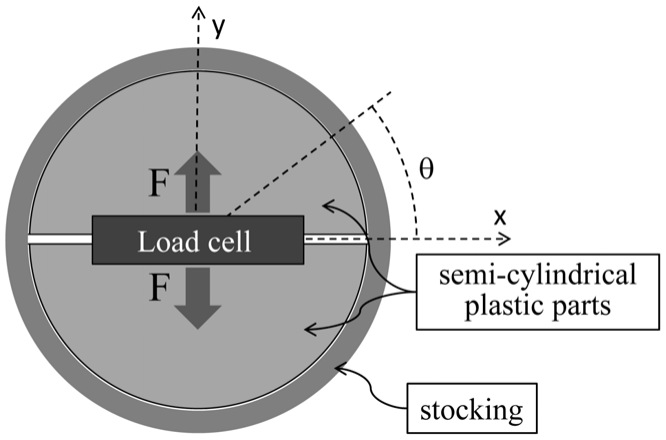
Schematic representation of a stocking stretched around the custom-made rig. Compression was derived from the measured force (F).

Stocking compression data were obtained at three sites (at the level of the malleoli [ankle], the widest point of the calf [mid-calf], and one inch below the top of the stocking [knee]). Compression measures were not conducted for the ankle placebo stocking, which terminated below the malleoli. A custom-made rig was used to measure compressive pressure in representative slices through a modeled calf at three locations, consisting of a load cell (Futek Advanced Sensor Technologies, Inc, Irvine, CA, USA, model LLB350) mounted between two semi-cylindrical plastic parts (Figure 1). The stocking was stretched around the rig and force measured by the load cell via a data acquisition board (National Instruments USB 6259). Custom software was used to process the acquired data (LabVIEW 2009, National Instruments).

The relationship between force (F) and pressure (P) exerted by the stocking was derived by integrating the component of pressure along the axis normal to the load cell (y-axis, Figure 1) on the interval [0, π]:
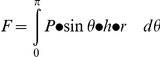
where *h* and *r* are the height and radius of the two semi-cylindrical plastic parts and *θ* is the angle represented in Figure 1. The pressure exerted by the stocking was computed from the measured force as follows:
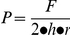



### Statistical analyses

Statistical analyses were performed using SigmaPlot version 11 (Systat Software Inc, San Jose, CA) and JMP (Statistical Analysis Systems, Cary, North Carolina.) Data were tested for normality using the Kolmogorov and Smirnov assumption and parametric or non-parametric testing used accordingly. Data are reported as means ± SEM. Significance was assumed where p<0.05. Comparisons between groups and over time were conducted using repeated measures ANOVA, with the Tukey or Bonferroni post hoc test. Differences in OT between conditions were determined using a randomised complete block design ANOVA. We also used a two-factor blocked analysis of variance to analyze the OT data, where stocking condition and order of intervention were the explanatory variables (factors) and the subject was the block. Correlations between variables were determined using Pearson Product Moment Analyses or Spearman Rank Order tests for parametric and non-parametric data respectively. Multiple regression analyses were used to develop a predictive model for the expected change in OT from selected anthropometric characteristics.

## Results

### Orthostatic tolerance

**Figure 2:**
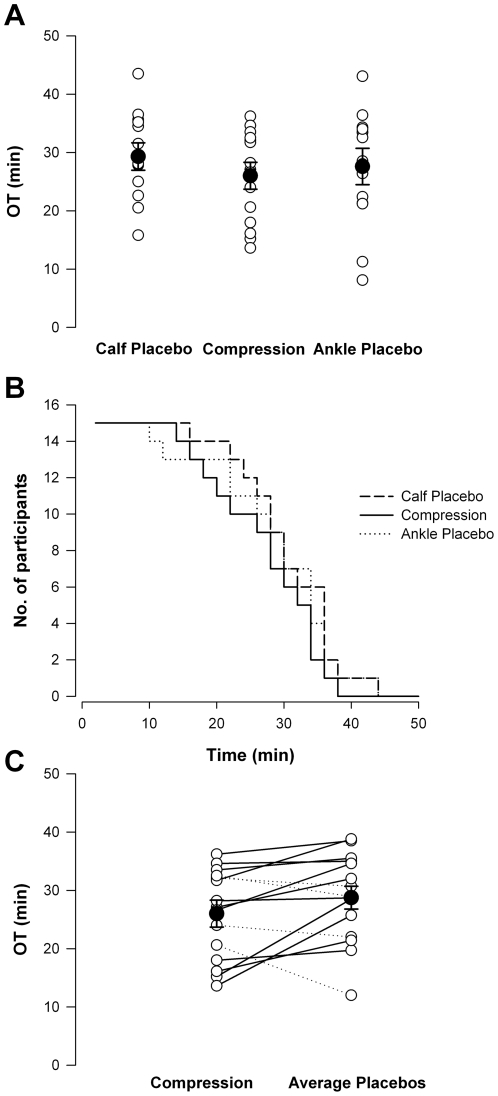
OT in the three test conditions. There were no significant differences in OT between conditions (A). Kaplan-Meier plots also revealed similar times to presyncope in all test conditions (B). When the two placebo conditions were combined, OT remained similar in placebo compared to compression stocking conditions (C). Dashed lines denote those in whom OT improved with compression stockings compared to placebo conditions, and solid lines denote those in whom OT was worse with compression stockings. Filled circles denote mean data.

All subjects experienced presyncope with hypotension, which triggered termination of each test, consistent with a vasovagal response. The time to presyncope was not significantly different between the three conditions (calf placebo 29.9±1.8, ankle placebo 27.6±2.4, and compression stocking 26.0±2.0 min; data for each experimental condition will be presented in this order throughout), Figure 2A. Kaplan-Meier plots also indicated no differences in OT on the three test days (Figure 2B). Therefore, we combined data from the two placebo conditions. The OT remained similar between the placebo and compression stocking conditions (Figure 2C). There was no significant effect of the order in which the interventions were received, and no significant interaction between the stocking condition and order in which the stockings were applied.

### Cardiovascular responses

#### Blood pressure

**Figure 3:**
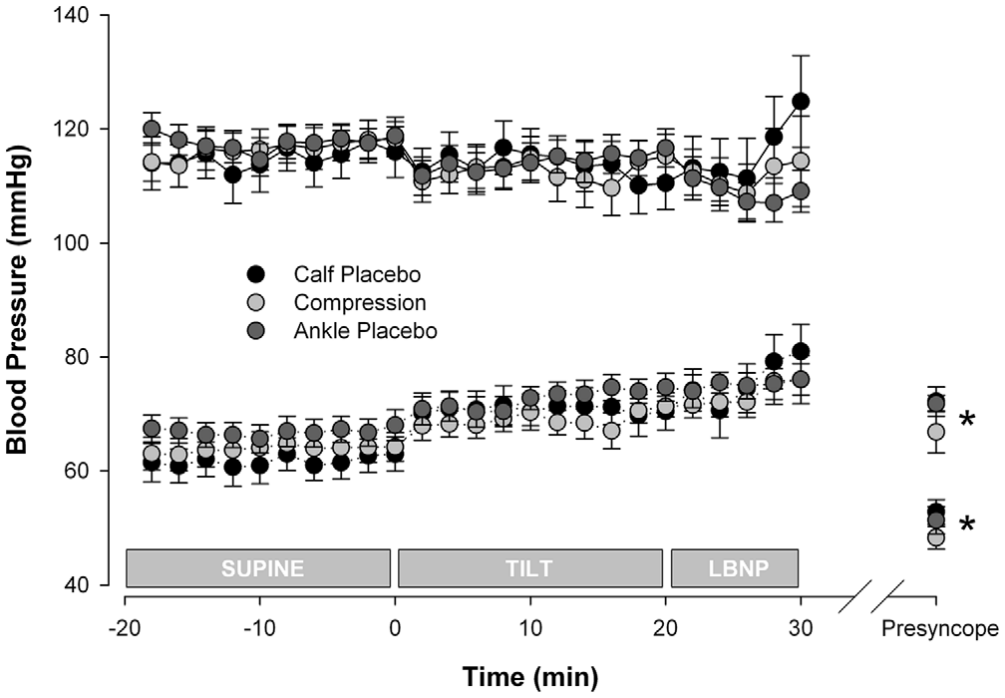
Blood pressure responses in the three test conditions. Solid lines, systolic arterial pressures; dotted lines, diastolic arterial pressures. There were no significant differences in systolic or diastolic arterial pressures between test conditions at any time point. Values at presyncope were significantly reduced compared to supine in all conditions (* denotes p<0.01).

Resting blood pressures were similar in all three conditions (114.9±4.1/61.7±3.0 mmHg, 116.4±3.3/63.8±2.6 mmHg and 117.6±2.5/66.8±2.3 mmHg). There were no significant differences in systolic or diastolic arterial pressures between conditions at any stage of testing (Figure 3). Blood pressure falls at presyncope were similar for all conditions (72.2±2.6/52.8±2.2 mmHg, 66.8±3.7/48.3±1.9 mmHg and 71.8±1.5/51.3±2.4 mmHg).

#### Stroke volume, heart rate, and cardiac output

**Figure 4:**
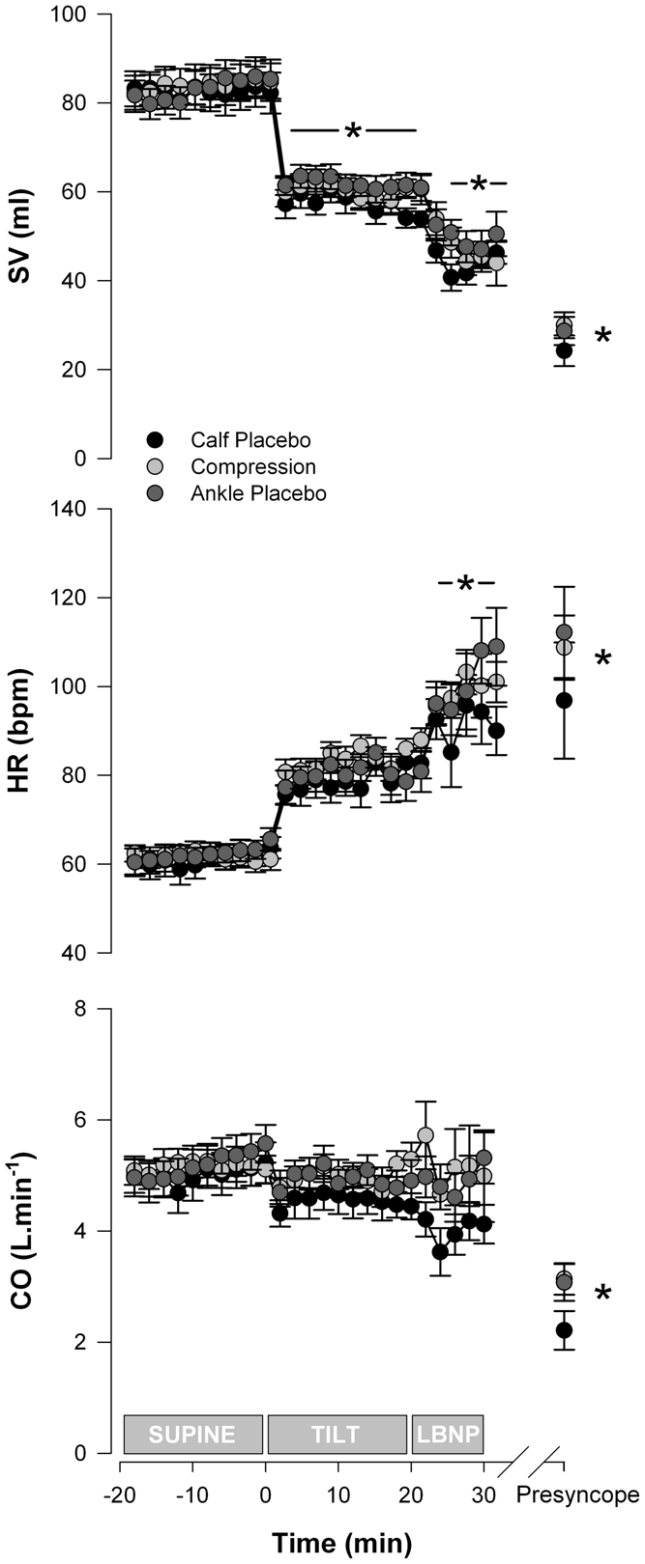
HR, SV and CO responses in the three test conditions. There were no significant differences in HR, SV or CO between test conditions at any time point. Significant differences from supine in all conditions are indicated by the * (p<0.05).

Supine SV were similar in all three conditions (82.6±4.2, 83.9±3.9 and 83.1±3.2 ml, Figure 4). Values at presyncope, during tilt and LBNP, were significantly reduced compared to supine (p<0.001).

Resting HR (61.2±2.7, 61.7±1.9 and 62.2±2.3 bpm), as well as the maximum HR responses to the orthostatic stress (115.9±5.5, 110.3±4.9 and 113.9±6.0 bpm), were similar in all conditions. Maximum responses, and values at presyncope and during LBNP, were significantly greater than supine in all conditions (p<0.01).

Resting CO were similar in all conditions (5.0±0.3, 5.2±0.3 and 5.2±0.3 L). Values at presyncope were significantly reduced compared to supine, tilt and LBNP (p<0.01).

There were no significant differences in SV, HR or CO between conditions at any stage of testing.

#### Peripheral resistance responses

Resting TPR (1527±121, 1422±70 and 1373±85 dyne.s^−1^.cm^−5^) and FVR (11.7±1.7, 10.5±1.6 and 13.6±2.1 units) were similar in all three conditions. There was a significant increase (p<0.05 compared to supine) in both TPR (maximum response 2492±419, 1983±200 and 1632±88 dyne.s^−1^.cm^−5^) and FVR (maximum response 30.1±4.9, 17.9±2.3 and 45.1±13.4 units) during orthostatic stress in each condition. The magnitudes of these responses were similar for each test condition.

There were no significant differences in TPR or FVR between conditions at any stage of testing.

#### Cerebral haemodynamics

**Figure 5:**
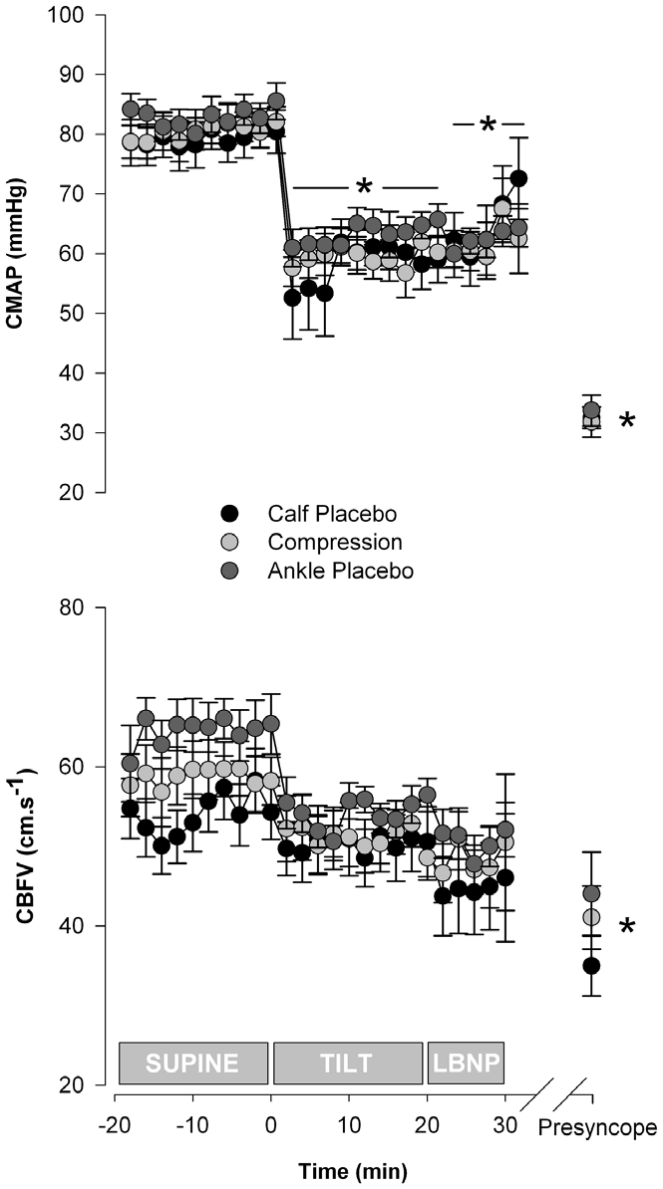
CMAP and CBFV in the three test conditions. There were no significant differences in CMAP or CBFV between conditions at any time point. Significant differences from supine in all conditions are indicated by the * (p<0.05).

CMAP was significantly reduced in all conditions during orthostatic stress compared to supine (Figure 5). In each condition, there was a further significant reduction in CMAP at presyncope compared to supine. Values at presyncope were similar in each condition. There were no significant differences in CMAP between conditions at any stage of testing.

CBFV was similar at rest in all conditions (54.4±3.5, 58.9±3.3 and 64.6±3.8 cm.s^−1^), Figure 5. CBFV was maintained, until presyncope, at levels not significantly different from supine for each condition. At presyncope, CBFV decreased compared to supine values, to 37.2±3.8, 41.0±4.0 and 44.0±5.2 cm.s^−1^ for each test respectively. The magnitude of the reduction in CBFV was similar for each condition (−19.8±4.2, −22.2±4.2 and −14.1±5.8 cm.s^−1^).

**Figure 6:**
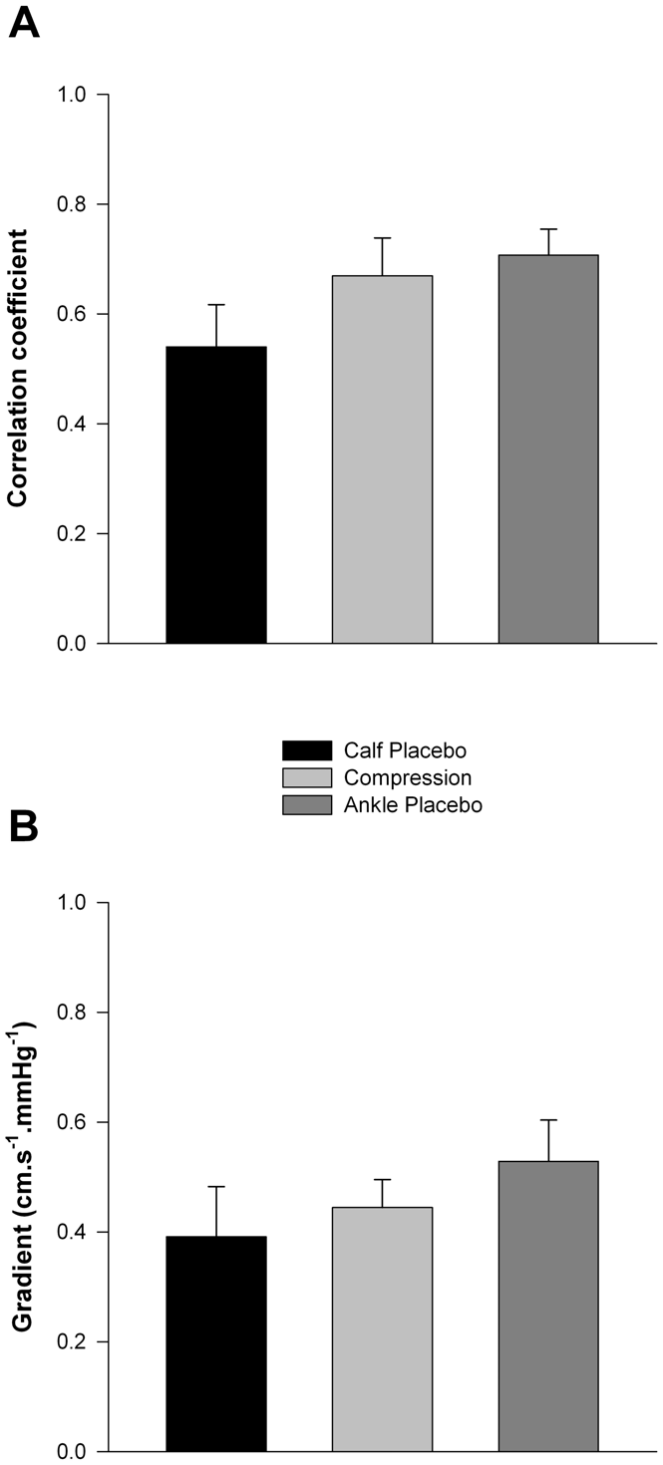
Cerebral autoregulatory responses in the three test conditions. There were no significant differences in either the correlation coefficient (A) or the gradient (B) describing the efficiency of cerebral autoregulation between the three conditions.

CVR was not significantly different between conditions at any stage of testing and did not change significantly within each test compared to supine. Both the correlation coefficient and the gradient describing the relationship between CMAP and CBFV were similar for all conditions (Figure 6).

#### End-tidal gases

There were no significant differences in P_ET_CO_2_ or P_ET_O_2_ between conditions at any stage of testing. The P_ET_CO_2_ decreased, and P_ET_O_2_ increased, at presyncope compared to supine in each condition (p<0.001), suggesting hyperventilation relative to baseline values. The absolute values were not significantly different at presyncope between conditions. The magnitude of the reduction in P_ET_CO_2_ from supine to presyncope was also similar in all conditions (−7.4±1.2, −6.6±1.0 and −7.0±1.1 mmHg).

### Relationships between orthostatic tolerance and anthropometric variables

**Figure 7:**
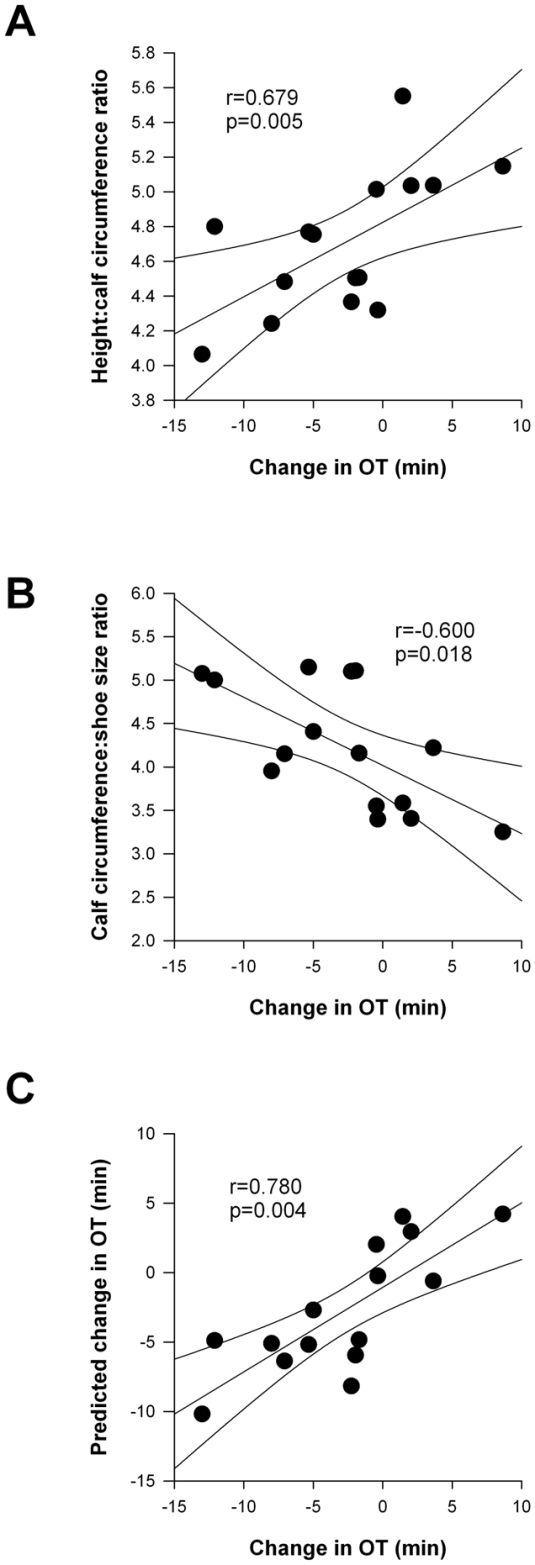
Relationship between the change in OT with compression stockings and anthropometric variables. There was a significant positive correlation between the change in OT and the height∶calf circumference ratio (A). There was a significant negative correlation between the change in OT and the calf circumference: shoe size ratio (B). The efficacy of the compression stockings could be predicted from the subject's shoe size and calf circumference (C).

Although the mean OT was not different between conditions, we noted considerable variability between individual responses, with some showing greater OT with compression stockings, and some showing reduced OT (Figure 2C). To examine whether this might be related to anthropometric variables, we qualified the OT while wearing the compression stocking relative to the mean of the two placebo conditions. The change in OT was positively correlated to the height∶calf circumference ratio and negatively correlated to the calf circumference∶shoe size ratio (Figure 7A & B). The efficacy of the compression stocking was predicted by a model based on the calf circumference and shoe size (Figure 7C) as follows:
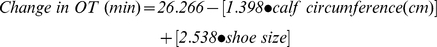



There was no significant relationship between the change in OT and the calf circumference measurements when expressed as muscle or adipose cross-sectional areas.

### Calf compression measurements

**Figure 8:**
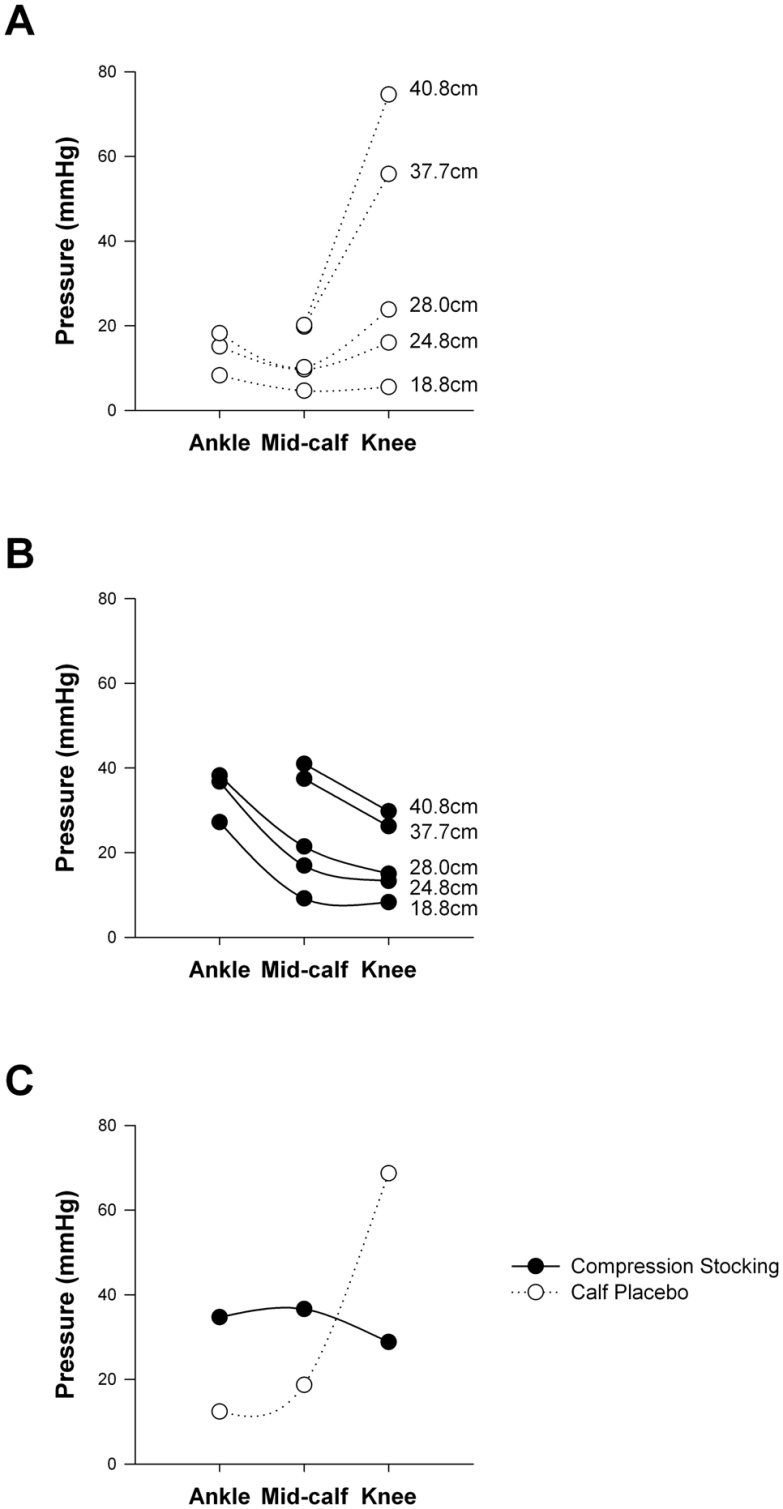
Compression levels for the compression and calf placebo stockings. Compression pressures applied over a range of distending circumferences can be seen at each region of interest for the calf placebo (A) and compression stockings (B). The compression pressures applied by the two stockings were compared at physiological distending circumferences for each region of interest (C).

Compression data could not be collected for the ankle placebo stocking. The calf placebo stocking applied minimal compression at low distending circumferences (Figure 8A), but a tight band at the knee resulted in high compression levels at this point with larger distending circumferences. The compression stocking applied graded compression at all distending circumferences, with the highest levels at the ankle, and lowest levels at the knee (Figure 8B). The measured calf circumference at the mid-calf in our volunteers was 37.1±0.8 cm (range 32.3–41.5 cm), similar to previous reports [32], [37]. Typical values for leg circumferences at the ankle and knee are 22.7±0.1 cm and 39.9±0.4 cm [32], [37]. When the two stockings were compared at these physiological distending circumferences for each region of interest (Figure 8C) it was seen that the compression stocking applied higher pressures at the ankle and mid-calf, but lower pressures at the knee compared to the calf placebo stocking.

## Discussion

We have demonstrated that graded calf compression stockings had no significant effect on OT in a randomised, double-blind, placebo-controlled study. Despite the lack of improvement in OT with compression stockings in the group as a whole, some individuals exhibited modest improvements in OT with compression stockings whereas others did not. From our anthropometric analysis we found that the calf circumference was a key determinant of the efficacy of the compression stocking. This has implications for their clinical use for the treatment of orthostatic intolerance, and underscores the need for individualised therapy when considering compression stockings as a treatment paradigm.

We selected a test that has a clearly defined end-point in all subjects, and has been shown previously to be highly reproducible, sensitive and specific [30], [31], [38]. As such, we are confident that had there been a significant effect of the compression stocking on OT we would have been able to detect it. We are also confident that the test end point (presyncope), and therefore the OT, was reliably determined because the terminating blood pressures and HR (as well as the other cardiovascular variables) were similar in all conditions. Furthermore, the investigator responsible for terminating the test was blinded to the test condition, to ensure this could not have influenced the result.

We tested a compression stocking reported to generate 20 mmHg compression at the ankle, graduated to 15 mmHg at the knee (www.sigvarisusa.com). At the mean leg circumferences of our group we measured compression of 35 mmHg at the ankle, graduated to 29 mmHg at the knee for this stocking, higher than quoted by the manufacturer. We found that the calf placebo may not have been a true placebo because although it applied minimal compression at the ankle and mid-calf (<15 mmHg) it did apply high compression just under the knee. However, we also included the ankle placebo, which could not have applied calf compression. Given that the responses were similar in all conditions, we are confident in our assertion of the lack of efficacy of the compression stocking tested. Finally, although this study was randomised, we also examined the possibility that there could be an effect or interaction between the order in which the stockings were tested and the orthostatic tolerance. This was not the case.

Our findings are compatible with earlier observations that compression of the abdomen is more effective than the calf for the improvement of OT [24]–[26]. However, some subjects had modest improvements in OT from calf compression stockings, and this could be predicted from simple anthropometric variables. The question as to why the anthropometric data influence the efficacy of the stockings remains. It could be that the compression stockings were over-stretched in those with large calf circumferences relative to their shoe size, applying higher pressures than intended. If sufficient to impede venous return, this could exacerbate venous pooling and so reduce OT. In contrast, in those with a smaller calf circumference relative to their shoe size, the compression may be just sufficient to enhance venous return, and delay the onset of syncope. Further studies are required to examine these possibilities. The fact that the efficacy of the compression stockings was related to the calf circumference, and not the proportion of muscle, suggests that this effect is not mediated via alterations in the mechanics of the skeletal muscle pump with compression stockings. However, we acknowledge that with tilt testing the skeletal muscle pumps are largely inactivated, so their potential role in the efficacy of compression stockings during active standing is unclear.

### Cardiovascular responses

Despite testing in healthy adults, six subjects exhibited poor OT during each test, compared to previously published “normal” values [31]. We and others occasionally observe poor OT in apparently healthy controls [39], [40], and this false positive response during tilt testing appears to reflect impaired reflex control of the circulation that is compensated by greater activation of the skeletal muscle pump during active standing [39], [41]. The fact that our control population included some individuals with poor OT does not negate our finding that compression stockings were ineffective at improving OT. In fact, it strengthens this argument, because the potential ceiling effect of testing only individuals with high OT is lessened. Furthermore, sub-analyses revealed that the influence of compression stockings was not related to the baseline OT.

Cardiovascular responses to the test were similar in all conditions at all time points. This observation underscores both the repeatability of the test, and the lack of efficacy of the compression stockings.

As expected in healthy controls, systolic and diastolic arterial pressures were maintained throughout testing, until the point of presyncope, reflecting appropriate arterial baroreflex responses to the gravitational fluid shifts imposed. At presyncope there was a sudden fall in arterial pressures, consistent with the onset of a vasovagal response [2], [21]. In all subjects each test was terminated with a systolic pressure below 80 mmHg, associated with symptoms of presyncope.

We observed baroreflex-mediated tachycardia that increased in a stepwise fashion at the beginning of each test phase [21]. The magnitude of this response was similar for all conditions. We did not observe significant bradycardia at presyncope in all subjects, likely due to either prompt termination of the test (prior to the bradycardia that typically accompanies a vasovagal response), or reflecting that this cardioinhibitory component of the reflex is not always present [42].

SV also decreased in a stepwise fashion during the orthostatic stress, decreasing by approximately 67% in all conditions at presyncope. This is compatible with reduced venous return when upright, secondary to venous pooling and plasma filtration [21].

CO was maintained throughout each test, until presyncope, when it decreased precipitously. The maintenance of CO prior to presyncope likely reflects the intact baroreflex response in these healthy control volunteers, whereby reductions in SV were accompanied by compensatory increases in HR. Indeed, the increase in HR in all conditions was approximately 73%, closely matching the fall in SV.

We observed baroreflex-mediated increases in FVR and TPR during orthostatic stress in each condition. This response was smaller in magnitude than has previously been observed in healthy control volunteers, presumably reflecting that some volunteers in this study had poor OT, and impaired vascular responses [21], [43], [44].

In each condition, due to the hydrostatic gradient imposed when upright, CMAP decreased similarly with the initial postural change, but was then maintained until presyncope. Despite the fall in CMAP, CBFV was maintained throughout each test indicating intact autoregulatory responses, until presyncope when the perfusion pressures were below the lower limit of autoregulation [45]. Indeed, when we quantified autoregulation from the correlation coefficient and gradient describing the relationship between CMAP and CBFV (whereby a steep gradient and high correlation coefficient indicate impaired autoregulation) [36] we confirmed similar autoregulatory control in each test condition. Accordingly, CVR responses were also similar between conditions. Again, this is compatible with minimal haemodynamic effect of the compression stockings. We also determined P_ET_CO_2_ and P_ET_O_2_ throughout testing, because of their known effect on CBFV [46]. Although P_ET_CO_2_ decreased and P_ET_O_2_ increased at presyncope, compatible with the modest hyperventilation that is known to accompany presyncopal episodes [46], the magnitude of these changes was similar for each test, confirming a similar challenge to cerebral autoregulation on each occasion.

Thus, the use of graded calf compression stockings did not influence cardiovascular responses during an orthostatic stress continued to presyncope.

### Limitations

We evaluated the efficacy of graded calf compression stockings on OT, and accordingly our results may not extend to other compression garments. The existing literature suggests that compression garments extending to the thigh and abdomen may be more effective at preventing orthostatic intolerance [24]–[26], but are associated with poor patient satisfaction and compliance [28], [29]. Future studies may wish to examine the optimum compromise between efficacy, comfort, and patient compliance.

Although subjects were not informed which stocking they were wearing on each test day, nor were they told the anticipated outcome of the test, it is possible that the study was not truly double-blinded. The ankle placebo is visually distinct from the calf placebo and compression stocking, and subjects may have been aware of different sensations of compression or tightness of the stockings. However, when questioned after completion of all three conditions, volunteers could not consistently identify the compression stocking.

Thirdly, we chose to conduct testing in healthy volunteers, and it is not known whether the results would extend similarly to patient populations. However, we expect this would be the case, because a number of our control volunteers actually had poor OT, similar to that of patients with syncopal episodes. Furthermore, other non-pharmacological approaches to prevent or delay syncope apply equally well to both patients and controls [47], [48].

Finally, it may be that the application of compression stockings prior to rising in the morning would have a greater effect, due to the “water jacket effect”, whereby oedema accumulating during the day restricts further venous pooling [49]. However, it has been shown that 20 minutes of supine rest is sufficient to normalise any prior venous pooling/capillary filtration effect [20], at least in control subjects, so we consider this unlikely.

### Conclusions

These data question the use of calf compression stockings for orthostatic intolerance and highlight the need for individualised therapy accounting for anthropometric variables when considering treatment with compression stockings.
